# Meta-analysis of genetic variants associated with human exceptional longevity

**DOI:** 10.18632/aging.100594

**Published:** 2013-08-24

**Authors:** Paola Sebastiani, Harold Bae1, Fangui X. Sun, Stacy L. Andersen, E. Warwick Daw, Alberto Malovini, Toshio Kojima, Nobuyoshi Hirose, Nicole Schupf, Annibale Puca, Thomas T Perls

**Affiliations:** ^1^ Department of Biostatistics, Boston University School of Public Health, Boston MA 02118, USA; ^2^ Section of Geriatrics, Department of Medicine, Boston University School of Medicine and Boston Medical Center, Boston, MA 02118, USA; ^3^ Division of Statistical Genomics, Washington University School of Medicine, St. Louis, MO 63110, USA; ^4^ Laboratorio di Informatica Biomedica, Dipartimento di Ingegneria Industriale e dell'Informazione, Università di Pavia, Pavia, Italy; ^5^ Research Center for Physical Fitness, Sports and Health, Toyohashi University of Technology, Toyohashi, Japan; ^6^ Division of Geriatric Medicine, Department of Internal Medicine, Keio University School of Medicine, Tokyo, Japan; ^7^ Taub Institute for Research on Alzheimer's Disease and the Aging Brain, Columbia University Medical Center, New York, NY 10032, USA; ^8^ Unit of Genetics, Cardiovascular Research Institute Istituto Ricovero Cura Carattere Scientifico Multimedica, Sesto S. Giovanni, Italy; Facolta' di Medicina, Universita' di Salerno, Baronissi, Italy

**Keywords:** centenarian, exceptional longevity, genetic association study, aging, gene, lifespan, meta-analysis

## Abstract

Despite evidence from family studies that there is a strong genetic influence upon exceptional longevity, relatively few genetic variants have been associated with this trait. One reason could be that many genes individually have such weak effects that they cannot meet standard thresholds of genome wide significance, but as a group in specific combinations of genetic variations, they can have a strong influence. Previously we reported that such genetic signatures of 281 genetic markers associated with about 130 genes can do a relatively good job of differentiating centenarians from non-centenarians particularly if the centenarians are 106 years and older. This would support our hypothesis that the genetic influence upon exceptional longevity increases with older and older (and rarer) ages. We investigated this list of markers using similar genetic data from 5 studies of centenarians from the USA, Europe and Japan. The results from the meta-analysis show that many of these variants are associated with survival to these extreme ages in other studies. Since many centenarians compress morbidity and disability towards the end of their lives, these results could point to biological pathways and therefore new therapeutics to increase years of healthy lives in the general population.

## INTRODUCTION

In Sebastiani et al “Genetic signatures of exceptional longevity in humans” [1], we presented the results from a genome wide association study of exceptional longevity in 801 centenarians from the New England Centenarian Study (NECS, mean age at death 104 years) and 914 genetically matched controls. The study identified a group of 281 SNPs that, used jointly in a genetic risk model, had 60% sensitivity to discriminate between centenarians and healthy controls. The sensitivity of the model however increased with more extreme ages of the centenarians and reached 85% for subjects age>107 years. The 281 SNPs included rs2075650 in TOMM40/APOE that reached irrefutable genome-wide significance and replicated in an independent cohort of 253 nonagenarians and centenarians from the Elixir Pharmaceuticals Study of Extreme Longevity and 341 genetically matched controls. The other 280 SNPs were statistically significant with p-values ranging between 10-2 and 10-6 although their level of significance did not meet the stringent criterion for genome-wide significance of 5x10-8, thus raising the possibility that these associations could be false positives. We therefore set out to determine which of these 281 SNPs were associated with longevity in a meta-analysis that included the two original studies, in addition to a case control study of longevity with nonagenarians and centenarians from the Southern Italian Centenarian Study [2], and a case control study of nonagenarians and centenarians from the Long Life Family Study [3]. We also extended the meta-analysis to include genotype data of a subset of SNPs from the Japanese Centenarian Study [4].

## RESULTS

Table 1 lists the studies' characteristics. The ELIX, SICS, LLFS and JCS case-control studies were all smaller than the NECS and cases in the ELIX, SICS and LLFS were younger than the NECS. Controls in the LLFS were males who died by the age of 94 or females who died by the age of 95, and 85% of these controls are relatives of the cases (eg siblings who died at younger ages) from the same family of the cases, so that they provide the strongest type of genetic matching. Some of the controls in the NECS and ELIX studies were chosen from the Illumina repository of controls and their ages are unknown.

**Table 1:** Description of the Studies

Study Population	Symbol	N Cases	Age of Cases	N Controls	Age of Controls	Genotyping Platform
Elixir Pharmaceutical Longevity Study	ELIX	253	100 (89-114)	341	NA	Illumina 370/550/610
Japanese Centenarian Study	JCS	513	106 (100-114)	561	69 (19-89)	Affymetrix 500KEA/500K/5.0
Long Life Family Study	LLFS	738	98 (95-110)	356	91 (44-95)	Illumina Omni 2.5
New England Centenarian Study	NECS	801	104 (95-119)	914	73 (53-90)^(1)^	Illumina 370/550/610/1M
Southern Italian Centenarian Study	SICS	410	95 (90-109)	553	NA	Illumina 317/370

Summary characteristics of the studies included in the meta-analysis. Samples genotyped with the Illumina 550 array are from the Illumina iControlDB. Controls in the NECS and ELIX studies were genetically matched as described in [1], controls in the SICS and JCS study were geographically matched, and 86% of controls in LLFS were family matched.

(1) Summary ages for 241 of the 914 controls enrolled in the NECS.

**Figure 1:**
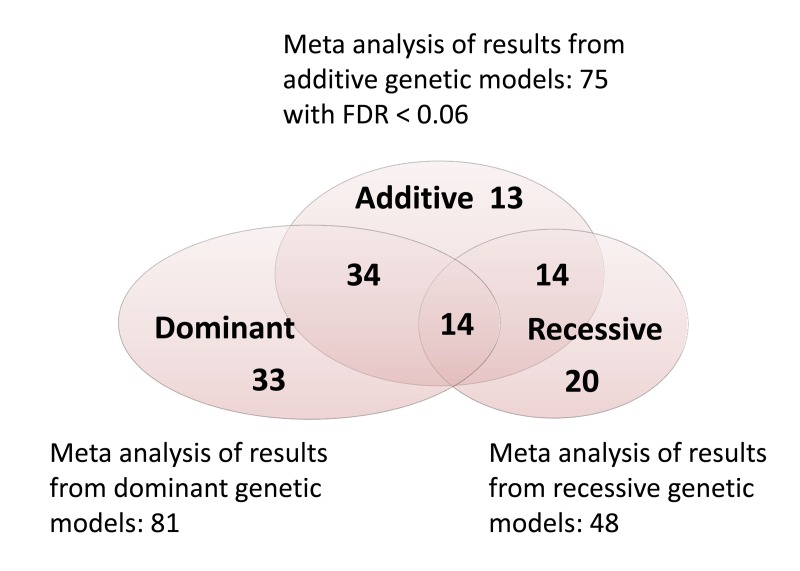
Venn diagram showing the number of significant associations from the meta-analysis of additive, dominant and recessive models when a 6% false discovery rate (FDR) was used. Genotypes were called using the top-strand rule and dominant and recessive models were coded for the top-strand allele A as explained in methods.

In the meta-analysis of additive genetic associations in NECS, ELIX, SICS and LLFS, 10 SNPs reached statistically significant association after Bonferroni correction (p-value < 0.05/280=0.00018). An additional 4 SNPs reached Bonferroni corrected statistical significance using a dominant model for the top-strand allele, and two SNPs reached Bonferroni corrected statistically significance using a recessive model for the top-strand allele (Table 2). The number of significant was much larger when a 5% and 6% false discovery rate corrections were used (Supplement Table 1). The Venn diagram in Figure 1 the number of significant SNPs from the meta-analysis of additive, dominant and recessive models, and 128 SNPs reached statistical significance with 6% false discovery rate. Note the substantial overlapping between the results with different genetic models. In fact, the 3 parameters of the additive, dominant and recessive models are functionally related and the tests are not independent. The full list of results for the meta-analysis of the 280 SNPs is in Supplement Table 1.

**Table 2:** SNPs that reached Bonferroni corrected significance in meta-analysis of results using additive, dominant and recessive models of Caucasian studies

Row	SNP	Gene	Alleles	A.AF^1^	CA^2^	NECS. OR	SICS.OR	ELIX.OR	LLFS.OR	MetaOR (95% CI)	pval
1	rs2075650	TOMM40/APOE	A/G	0.925	G	0.492	0.726	0.499	0.507	0.527 (0.452;0.616)	4.44E-16
2	rs1525501	NA	A/G	0.128	G	0.761	0.836	0.510	0.719	0.724 (0.630;0.831)	4.94E-06
											
3	rs3803833	NA	A/C	0.875	C	0.712	0.974	0.856	0.667	0.765 (0.675;0.867)	2.72E-05
4	rs1016013	NA	A/G	0.373	G	1.300	1.168	1.186	1.089	1.206 (1.105;1.317)	2.87E-05
											
5	rs216148	CSF1R	A/G	0.124	G	0.692	1.011	0.550	0.798	0.753 (0.655;0.865)	6.36E-05
6	rs1867102	C9orf3	A/G	0.477	G	1.295	1.144	1.107	1.117	1.195 (1.094;1.304)	7.28E-05
7	rs1822590	NA	A/C	0.277	C	1.309	1.120	1.109	1.190	1.208 (1.100;1.327)	7.57E-05
8	rs4918255	SORCS1	A/G	0.665	G	1.265	1.103	1.180	1.238	1.212 (1.101;1.333)	8.81E-05
9	rs1456669	NA	A/C	0.160	C	0.638	0.835	1.118	0.817	0.777 (0.685;0.882)	9.89E-05
10	rs915179	LMNA	A/G	0.541	G	1.342	1.112	1.198	0.999	1.191 (1.090;1.301)	0.00010
											
11	rs2738679	WWOX	A/G	0.700	GG	1.840	1.213	1.802	1.587	1.600 (1.270;2.017)	6.85E-05
12	rs17702471	GPC6	A/G	0.785	GG	2.512	1.673	1.613	1.142	1.810 (1.347;2.431)	8.33E-05
13	rs1042663	C2	A/G	0.109	GG	0.617	0.808	0.629	0.988	0.720 (0.612;0.848)	8.386E-05
14	rs651922	DCPS	A/G	0.724	GG	2.312	1.035	2.040	1.079	1.650 (1.270;2.145)	0.00018
15	rs11218921	NA	A/G	0.920	AG/GG	0.502	0.655	0.810	0.523	0.590 (0.457;0.762)	5.47E-05
16	rs2738173	DEFB1	A/G	0.842	AG/GG	0.652	0.793	0.973	0.945	0.778 (0.683;0.887)	0.00016

Sixteen SNPs that reached Bonferroni corrected statistical significance (0.05/281=0.00018) in the meta-analysis of additive models (rows 1–10); dominant models for the A allele (rows 11–16) and recessive model for the A allele (rows 15-16).

1 A.AF= frequency of A allele in NECS controls

2 CA= coded allele in genetic models.

Only 19 SNPs in the set of 28 that reached FDR corrected significance had genotype data available in the JCS set, and meta-analysis of these 19 SNPs was extended to include the results for the JCS set Six SNPs (see Table 3) reached Bonferroni corrected significance (p-value< 0.0026=0.05/19) in the meta-analysis of results from additive models (4 SNPs), dominant models (1 SNP), and recessive models (1 SNP) and 4 of these 6 SNPs were not included in the list of 16 that reached Bonferroni corrected significance in the meta-analysis of NECS, ELIX, SICS and LLFS. Fourteen SNPs in 19 reached 6% FDR corrected statistical significance. Full details of the analysis that included the JCS set is in Supplement Table 2.

**Table 3:** SNPs that reached Bonferroni corrected significance in meta-analysis of results using additive, dominant and recessive models of Caucasian and Japanese studies

Row	SNP	Gene	Allele	A.AF^1^	CA^2^	NECS.OR	SICS.OR	ELIX.OR	JCS.OR	LLFS.OR	MetaOR	pval
1	rs1525501	NA	A/G	0.128	G	0.761	0.836	0.510	0.949	0.719	0.807 (0.725;0.898)	8.97E-05
2	rs1456669	NA	A/C	0.160	C	0.638	0.835	1.118	0.937	0.817	0.825 (0.743;0.916)	0.000312
3	rs4729049	CDK6	A/G	0.889	G	1.357	1.170	1.132	1.201	1.085	1.212 (1.078;1.362)	0.001311
												
4	rs11954180	SLC6A7	A/G	0.058	G	1.540	1.214	1.401	0.915	1.022	1.302 (1.105;1.535)	0.001639
												
5	rs2596230	RYR3	A/G	0.876	GG	4.175	2.755	3.099	1.099	0.893	2.607 (1.536;4.423)	0.000383
6	rs1800392	WRN	A/C	0.481	AC/CC	0.636	0.815	0.923	0.898	0.906	0.787 (0.685;0.904)	0.000708

SNPs that reached Bonferroni corrected statistical significance (0.05/19=0.0026) in the meta-analysis of additive models 1-4); dominant models for the A allele (row 5), and recessive models for the A allele (row 6).

^1^ A.AF= frequency of A allele in NECS controls

^2^ CA= coded allele in genetic models.

**Figure 2:**
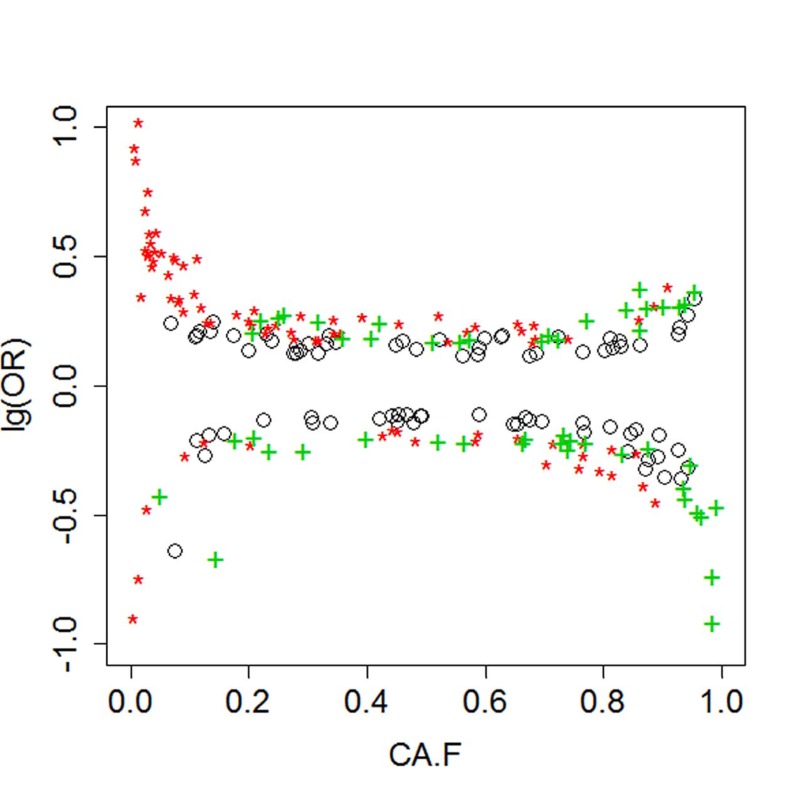
Genetic effects versus allele frequency Black circles= additive effects; Red asterisks: dominant effects; Green crosses= recessive effects.

The list of 28 SNPs include both common and uncommon variants that are associated with increase odds for extreme longevity. Figure 2 plots of genetic effects for different genetic models versus the frequency of coded alleles and shows that both uncommon variants (allele frequency < 0.05 or > 0.95) and common variants are associated with increased odds for longevity. Common variants tend to have more modest effects than rare variants.

**Figure 3:**
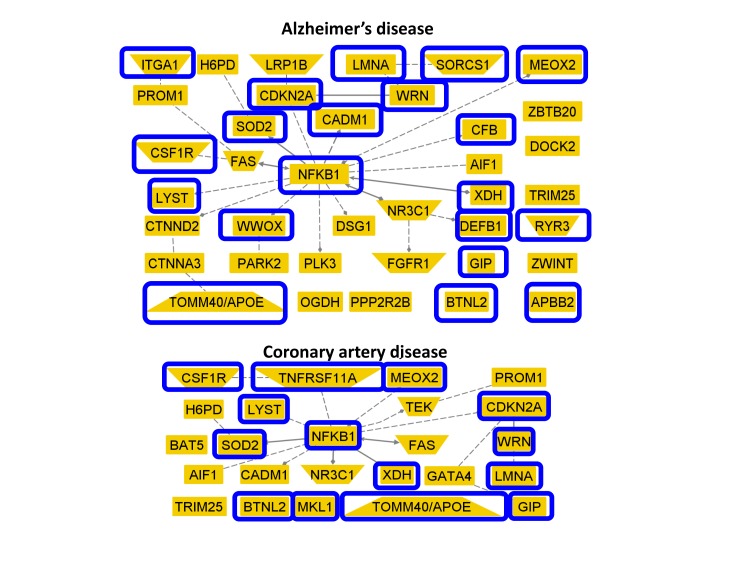
Genes with SNPs that reach statistical significance with meta-analysis and were implicated in Alzheimer's and coronary artery disease The two networks display 38 genes linked to Alzheimer's disease (top) and 24 genes linked to coronary artery disease (bottom) that included SNPs in the list of 281 in [1]. Genes circled in blue include SNPs that reached statistical significance in the meta-analysis.

Figure 3 shows two networks of genes associated with Alzheimer's and coronary artery disease that included SNPs in the list of 281 reported in Sebastiani et al [1]. Nodes circled in blue in the Figure show the genes that include SNPs reaching statistical significance in the meta-analysis (20 of 38 genes associated with Alzheimer's disease, and 14 of 24 genes associated with coronary artery disease).

## DISCUSSION

After the publication of the article by Sebastiani et al [1] that presented the corrected results from an article published online in Science Express and then retracted because of technical errors [5], there was discussion about the validity of the 281 SNPs associated with exceptional longevity (http://blog.23andme.com/news/studies-of-extreme-longevity-extremely-challenging/) and confusion about the correct SNP list associated with exceptional longevity [6]. The main objective of this article was therefore to cast some light on the validity of the individual associations of the 281 longevity SNPs. The meta-analyses of 5 case control studies of exceptional longevity of Caucasians and Japanese identified 0 SNPs that reached Bonferroni corrected significance, and 128 that reached statistical significance with a 6% false discovery rate. Besides arbitrary thresholds for statistical significance, the concordance of genetic effects for many of the SNPs across the 5 different studies is also providing evidence of true associations (See Tables 2 and 3, and the supplementary tables for SNPs that reached significance with 5% FDR correction). The large number of replicated SNPs provides further support for the hypothesis that exceptional longevity is a multifactorial trait that is determined in part by an enrichment for common genetic variants imparting protective effects [1, 7, 8]. The rare existence of families that demonstrate clustering for extreme age also suggests the existence of rare variants as well [9].

The list of 128 SNPs include common and uncommon variants that are associated with increased odds for exceptional longevity (Figure 2). The common variants have modest effects and although they reach statistical significance when the multiple correction penalty is limited to 280 tests, they would fail to reach statistical significance at genome-wide level. This result is consistent with other genetic studies of aging and longevity that failed to discover SNPs reaching genome wide significance even with substantially larger sample sizes [10-13], although these variants can be predictive of lifespan [14]. Some of the variants in Tables 2 and 3 are associated with decreased odds for exceptional longevity. It is interesting to note that lack of deleterious variants is not a major feature of these genetic data and we and others indeed showed that centenarians do not appear to differ in the number of deleterious variants compared to healthy controls [1, 15, 16].

Some of the SNPs listed in Tables 2 and 3 are in genes previously associated with underlying mechanisms of aging and age related diseases, and Figure 3 shows an example of replicated genes previously associated with Alzheimer's disease and coronary artery disease. The best known longevity variant in Table 2 is rs2075650 in TOMM40 and linked to APOE. This result is consistent with those reported by other studies [6,11] and by the recent linkage analysis in [17]. The SNP rs2075650 was linked to the ∊4 allele of the APOE gene although the strength of linkage disequilibrium between rs2075650 and the SNPs rs7412 and rs429358 that define the 3 alleles of APOE seems to vary with ethnicity. The Leiden Longevity Study reported a r2=0.56 between rs2075650 and rs429358 in 8946 subjects [11], while in 4576 subjects of the LLFS the r2 between rs2075650 and rs429358 was only 0.27 [18]. The association between the SNP rs2075650 and the ∊4 allele is stronger at the genotype level and in LLFS subjects, 88% of carriers of the GG genotype carried at least one copy of the ∊4 allele, compared to 73% prevalence of the ∊4 allele in carriers of the AG genotype and 6% in carriers of the AA genotype. Interestingly, the effect of the G allele was more modest in the SICS set. This result is also consistent with the known variability of APOE alleles with ethnicities [19]. Unfortunately SNP data were not available for assessment of this association in the JCS set. Note that SNPs in the Alzheimer's disease associated genes CEACAM16, CSFR1, SORCS1, WWOX and DEFB1 (Figure 3) also reached Bonferroni corrected significance.

The SNP rs915179 in LMNA showed consistent effects in the NECS, ELIX and SICS sets but not the LLFS set, although the association reached Bonferroni corrected statistical significance in the meta-analysis of Caucasian studies. This result is consistent with recent findings in [20] and the data included in this analysis partially overlap with those included in [20]. The synonymous coding SNP rs4641 is in moderate LD with rs915179 and we noted that two supercentenarians (ages at death > 114 years) were both heterozygous carriers of this mutation [21] which was associated with survival advantage as well as increased chance for metabolic trait. One study suggested that this advantage is related to increased body mass index (BMI) [22] that in the very old could compensate the effects of sarcopenia and osteoporosis. Supporting an important role of BMI and longevity is the significant association of the SNP rs3906146 in *LMX1B* and rs9899404 in *GIP* (Supplement Table 1, FDR < 6%). The SNP rs3906146 is in moderate LD with the less common SNP rs867559 (r2=0.32, D'=1, based on CEPH from HapMap) that was found associated with high BMI and obesity in [23]. These results are in agreement with an important role of insulin and longevity [24,25]. Two SNPs in the WRN gene reached statistical significance in the meta-analysis of Caucasian studies (rs3024239 and rs1800392, Supplement Table 1, FDR < 6%). The association of rs1800392 became significant at 5% Bonferroni corrected significance when the meta-analysis included the JCS set (Supplement Table 2).

The G allele of SNP rs2596230 in RYR3 (Table 3) showed consistent recessive effects in all the studies (Meta OR=2.61). This SNP has been associated with age-related macular degeneration [26]. Note that Table 2 includes an additional SNP (rs216148 in CSF1R) that replicates in the ELIX set that has also been linked to age-related macular degeneration [27].

Limitations. Although this study represents the largest meta-analysis of exceptional longevity to date, the overall sample size is still relatively small to capture small genetic effects. The LLFS set is very large compared to the other studies, but most of the participants are still alive and younger and the LLFS will become much more powerful over time as these individuals achieve older ages. In addition, the median ages at death in the ELIX, SICS and LLFS sets were younger than the median age at death of 104 years in centenarians of the NECS and this difference in ages as well as birth cohort differences may have limited the replication rate. An additional limitation is that although we included studies of different ethnicities and race, the JCS samples were genotyped with a different genotyping platform and imputation failed, so that the majority of the SNPs in the list of 281 could not be tested and additional genotyping and sequencing are necessary. Additional replication studies will provide further evidence in support of these and perhaps other associations.

## MATERIAL AND METHODS

### Ethic Statement

The NECS protocol is approved by the Boston University Medical Campus Institutional Review Board. The ELIX enrollment protocol was approved by the Western Institutional Review Board. The SICS study protocol is overseen and approved by the Multimedica Hospital Institutional Review Board in Milan (Italy). The JCS study protocol is approved by the Ethical Committee of Keio University School of Medicine. Participants of the LLFS underwent informed consent and the study was approved by the Institutional Review Boards of Boston University Medical Campus, University of Pittsburgh, Columbia University and Washington University St Louis, and the Ethics Board of the University of Denmark.

### Study Populations and Genotype Data

All studies are summarized in Table 1.

### The NECS case-control

set of 801 centenarians and 914 genetically matched controls is described in [1]. The study included centenarians from the NECS [28] (median survival 104 years, age range 95-119) and a combination of controls from the NECS (median age 73 years, age range 50-93) and from the Illumina control database (iControlDB). Ages were unknown for the majority of Illumina controls. The algorithm for genetic matching was described in [29]. The data were genotyped using Illumina arrays 370, 610 and 1M, and the final list of 281 SNPs passed stringent quality control measures, including a sample call rate >96% and a SNP call rate >98% in all array types. For independent validation of the genotype data, the top 30 SNPs included in the list were also genotyped using the TaqMan, with a concordance >99%. All details are described in [1] and its online supplement material.

### The Elixir study

also described in [1], included 253 long lived individuals (median survival 100 years; age range: 89-114) and 341 genetically matched controls. The long lived individuals were enrolled by Elixir Pharmaceuticals between 2001 and 2003 using a protocol that matched the NECS and samples were genotyped using the Illumina 370 and 610 arrays. The genetically matched controls were selected from the Illumina control database (iControlDB) using the same algorithm used for the NECS data. This study had a slightly larger enrichment of subjects with Eastern European, Ashkenazi Jewish ancestry compared to the discovery set.

### The Southern Italian Centenarian Study (SICS)

included 410 nonagenarians and centenarians (median survival 96, age range 90-109) and 553 geographically matched controls. The nonagenarians and centenarians were recruited by the Institute Longevita in Italy, beginning in 2003, using recruitment and data collection modeled after the NECS protocol. All DNA samples were genotyped using Illumina 317 and 370 arrays and the data were described in [2].

### The Japanese Centenarian Study (JCS)

included 513 long lived individuals (median survival 106 years; age range 100-114) and 561 geographically matched controls (median age 75 years; range: 26-89). The JCS enrolls Japanese centenarians from throughout Japan with an emphasis upon recruiting semi-supercentenarians (age 105+ years) and referent cohort subjects comprising spouses of centenarians' offspring and healthy younger volunteers. DNA samples were genotyped using the Affymetrix 500KEA, 500K, and 5.0 arrays with protocols described in [4].

### The Long Life Family Study (LLFS)

enrolled families enriched for longevity via 4 field centers (Boston, New York, and Pittsburgh in the USA, and Denmark) between 2006 and 2009 and participants are followed annually to update vital, medical and functional status. The recruitment protocol, described in [3], used the Family Longevity Selection Score (FLoSS) for an objective measure of familial longevity [30] and enrolled 583 families with a FLoSS > 7 consisting of 1493 probands, their siblings and 192 spouses in the older generation, and 2437 offspring and 809 of their spouses. Available to this replication study were genome wide genotype data from 4,567 subjects generated from the Illumina Omni chip 2.5 (2.5M SNPs), imputed to the 1000 genomes using MACH as described in [31]. The genotyping protocol, quality control and quality assessment included use of the GRR program to validate familial relations [32], the program Loki to validate Mendelian consistency [33], SNP call rate > 98% and sample call rate > 97.5%. LLFS participants have been followed since 2006 and 822 mortality events were noted (median age at death 95 years, range 44 −110) since enrollment up to April, 2013. We identified 738 subjects with age at last contact >94 years for males, and > 95 years for females matching the age threshold for definition of case in the NECS, and 356 subjects who died at younger ages and used these subjects for a case control study of extreme longevity.

### SNP selection

All 281 SNPs were available in the ELIX and SICS sets. We identified 209 of the 281 SNPs in the Illumina Omni arrays used for genotyping the DNA samples of the LLFS subjects, while good quality imputed data were available for the other 71 SNPs (mean r2=0.99), and one SNP could not be included in the analysis. The JCS DNA sample set was genotyped with Affymetrix arrays and since imputation did not yield reliable data, only 32 SNPs were available for replication after removal of SNPs with 0 genotype counts.

### Analytic approach

A meta-analysis of results was conducted using the rmeta package in R 3.01. Standard estimates of log-odds ratios and standard errors were calculated using allelic, dominant and recessive models for the top strand allele in NECS, ELIX, SICS and JCS case control studies. Continuity correction was used to estimate the log-odds ratios. SNPs alleles (A, B) were coded according to the top-strand rule, so the alleles of each SNP are either (A, G) or (A, C). Based on these alleles, dominant models for the A allele were defined by coding the 3 genotypes as AA/AB=0, versus BB=1, while recessive models for the A allele were defined by coding the 3 genotypes as AA=0 versus AB/BB=1. Log-odds ratios and standard errors were estimated using a mixed effect logistic regression model in LLFS case control study, adjusted for sex and significant genome-wide principal components that were estimated using eigenstrat [34]. The analysis of mixed effect logistic regression for the 3 genetic models was conducted in the OpenBUGs software, using vague prior distributions and normally distributed random effects to account for the within family relation of consanguineous subjects. The log-odds ratios of the 5 case control studies were meta-analyzed using inverse variance weights. Woolf's test of heterogeneity was used to decide upon fixed or random effects meta-analysis on a per-SNP base, and random effect meta-analysis was conducted for those SNPs in which the significance p-value of the test for heterogeneity was < 0.05/280. Stringent statistical significance was based on Bonferroni correction (0.05/280=0.00018 in meta-analysis of NECS, ELIX, SICS and LLFS results, and 0.05/21=0.0024 when the analysis was extended to include 21 SNPs that reached significance in the combined NECS, ELIX, SICS and LLFS studies and were available for analysis in the JCS case/control study). The Bonferroni correction is however unnecessarily stringent because it assume a 0% true positive rate in the set of 280. We therefore also used a 0.05 FDR correction using Benjiamini Hochberg algorithm [35].

## Conclusions

The original GWAS of exceptional longevity in centenarians from the NECS and genetically matched controls generated a substantial list of genetic candidates for human longevity that included both novel and well established longevity associated genes. The large number of SNPs that reached statistical significance in this analysis shows that many of these variants are robust and we expect to be able to replicate even larger numbers as studies grow in size, their subjects become older and hopefully many more studies will coordinate their efforts to use similar genotyping platforms so that a greater number of markers are comparable. Genetic variations associated with exceptional longevity that are noted in populations with very different genetic backgrounds may be particularly interesting and important to identify biologic pathways that influence exceptional longevity and processes as basic as rate of aging. Although some of the variants in Tables 2 and 3 are known to be associated with aging or age-related diseases, some of the replicated results point to novel genes that may influence aging and extreme lifespan and open new avenues of research on the genetic of extreme longevity.

The challenge is how to follow up these current results. Our multivariate modeling of exceptional longevity in [1] showed that different combinations of the 281 SNPs alleles determine different probabilities of survival to very old ages. Functional experiments that try to assess the individual effects of these variants with longevity may fail or be suboptimal because they would ignore other interacting genetic variants. A systems-based approach will be necessary to discover the synergistic and antagonistic effects of these many variants and their roles in extending lifespan and health-span.

## SUPPLEMENTARY TABLES




